# Soft skills and their relationship with life satisfaction and cognitive reserve in adulthood and older age

**DOI:** 10.1007/s10433-024-00820-2

**Published:** 2024-09-09

**Authors:** Tommaso Feraco, Nicole Casali, Elena Carbone, Chiara Meneghetti, Erika Borella, Barbara Carretti, Veronica Muffato

**Affiliations:** 1 Department of General Psychology, University of Padova, Via Venezia, 8, 35131 Padua, Italy; 2 Max Planck Institute for the Study of Crime, Security and Law, Freiburg, Germany

**Keywords:** Twenty-first century skills, Soft skills, Cognitive reserve, Life satisfaction, Aging

## Abstract

Soft skills are key factors for success in multiple contexts of daily life, as well as for life satisfaction, but little is known about their role in late adulthood and older age. In this study, we aimed to investigate the relationships of soft skills, as defined by the World Economic Forum, with two indicators of successful/healthy aging: life satisfaction and cognitive reserve. A sample of 435 adults aged 50 and over completed self-reported measures of soft skills, life satisfaction, and cognitive reserve. As control, fluid (reasoning) and crystalized (vocabulary) intelligence were assessed too, along with socio-demographic characteristics. A series of regression analyses showed that soft skills were positively related to both life satisfaction and, to a lower extent, cognitive reserve, above and beyond gender, age, and both fluid and crystallized intelligence. Interestingly, these associations were independent from participants’ age. Overall, these results highlight the importance of considering soft skills also over the middle-late adult life course, due to the potential role of these individual qualities in supporting an individual’s well-being and an active and engaged lifestyle, with implications for the promotion of a healthy aging.

**Supplementary Information:**

The online version contains supplementary material available at 10.1007/s10433-024-00820-2.

## Introduction

Soft skills are acquirable individual qualities regulating thoughts, emotions, and behaviors across various life contexts, facilitating the achievement of goals and fostering satisfying interpersonal relationships (Heckman and Kautz 2012; Park et al. 2004; Robles 2012; Soto et al. 2021). Recognized by several international bodies (European Commission 2016; World Health Organization 2003) as crucial abilities, they play a pivotal role in distinguishing individuals who succeed from those who struggle to do so, particularly in educational and organizational settings (Duckworth and Quinn 2009). Consequently, the World Economic Forum (WEF; World Economic Forum 2016) has introduced a framework outlining the most essential skills to navigate the ever-changing work environment and its intricate challenges. These soft skills include curiosity (being open-minded and inquisitive); initiative (proactively undertaking new goals and tasks); persistence (maintaining interest and effort to accomplish a task or goal); adaptability (adjusting oneself in light of new information to face new and uncertain situations); leadership (effectively guiding and inspiring others to accomplish a common goal); and social and cultural awareness (interacting with other people in a socially, culturally, and ethically appropriate way). Soft skills exert influence on achievement, work success, and life satisfaction, sparking increasing interest in incentivizing their development among youth and workers (Bruna et al. 2019; World Economic Forum 2016). But could they also sustain people through their older life? Only a few studies have examined this intriguing question.

This is particularly important because, as we age, there are changes that might impact our well-being and cognitive functioning (e.g., Borella et al. 2017; Craik and Salthouse 2011), and the identification of—new—factors related to healthy aging is still a priority (Curtis et al. 2015). Therefore, it is worthwhile to investigate the connection between soft skills, life satisfaction, and cognitive reserve, serving as proxies for well-being and lifestyles behaviors favoring an active aging (e.g., Borella et al. 2023). Indeed, both life satisfaction and cognitive reserve may be crucial for ensuring autonomy and quality of life of older adults. Revealing such associations could offer insights for future policies and interventions that target soft skills as positive correlates of healthy aging.

### Soft skills and life satisfaction

Life satisfaction refers to the subjective overall contentment with one’s life conditions (Diener 1984), and there is compelling evidence indicating a positive relationship between soft skills and life satisfaction in adults (Bruna et al. 2019; Weigold et al. 2020). However, this association may vary depending on the age considered. Studies suggest that the impact of persistence and social abilities on life satisfaction may slightly diminish with aging (Baumann et al. 2020). Contrarily, among soft skills, curiosity exhibits a stronger connection to life satisfaction in retired older adults compared to employed participants, as well as in those living alone compared to married individuals (Baumann et al. 2020).

There is also evidence suggesting that soft skills collectively form a unique second-order factor (Casali et al. 2022; Feraco et al. 2022a, b), and that this factor primarily relates to life satisfaction (Feraco et al. 2023a, b), while the specific variations and effects of individual skills may be negligible. This highlights the importance of evaluating such common factor also in older adults and specifically test whether such factor, and not the specific skills, can be predictive of life satisfaction. Thus, given that soft skills appear so relevant for life satisfaction (Soto et al. 2021) which is so crucial for quality of life in aging, it is fundamental to deepen the effect of soft skills (also as a general factor) on life satisfaction in adulthood and older age. However, no studies have systematically addressed such an issue.

### Soft skills and cognitive reserve

Cognitive reserve, the dynamic and active process to preserve appropriate functioning and behavior despite the presence of either typical age-related cognitive changes or neuropathological conditions (e.g., Stern 2009), represents a good candidate for the promotion of a healthy—cognitive—aging. It is related to a series of lifestyle-related socio-behavioral indicators (see Borella et al. 2023), linked to a stimulating and “enriched” environment that enables people to counteract age-related worsening in cognitive abilities and everyday functioning (Opdebeeck et al. 2016; Valenzuela and Sachdev 2006) and depressive mood (e.g., Borella et al. 2023; Handing et al. 2022), but also to promote life satisfaction (Fastame 2021; Tymoszuk et al. 2020).

Only a limited number of studies have sought to comprehend the connection between soft skills and cognitive reserve proxies in middle adulthood and older age. Baumann et al. (2020) found that social relationships skills (love or teamwork) are positively linked to living together with a partner compared to living alone, as well as being in a partnership versus being widowed (Baumann et al. 2020). Conversely, creativity and curiosity were shown to be associated with increased involvement in leisure, cultural/art activities, creative occupations, and new learning (Bourgault 2019; Palmiero et al. 2016). However, these studies neither embraced a soft skills framework with clear theoretical foundations nor operationalized cognitive reserve as a dynamic, multi-dimensional construct.

Overall, there is thus only initial evidence of an association between soft skills, life satisfaction, and cognitive reserve in middle adulthood and older age.

### Rationale for the study and hypotheses

This study undertook a novel examination of soft skills using a grounded and integrative framework, as delineated by the WEF (i.e., adaptability, curiosity, leadership, initiative, perseverance, and social and cultural awareness; 2016), in middle adulthood and older age (ranging from 50 to over 80 years). As these skills might together—as a unique factor—prompt individuals to adopt functional, proactive, and positive approaches to life challenges and situations, while the specificity of each skill could be negligible or only beneficial for smaller, specific tasks, or situations (Feraco et al. 2023a, b), we tested the tenability of such a single factor in older adults. We also examined whether soft skills are associated with life satisfaction and cognitive reserve, among the key factors for successful/healthy aging. Cognitive reserve was operationalized considering its multi-dimensional, life stage-dependent nature assessed with the current and retrospective cognitive reserve survey (Borella et al. 2023).

Therefore, this paper had three main aims. Firstly, it sought to investigate in middle adulthood and older age: (i) whether soft skills constitute a single factor (Aim 1); (ii) the associations of soft skills with life satisfaction and cognitive reserve (Aim 2); (iii) whether these relationships change across the adult life span, from middle adulthood to older age (Aim 3). Given the dynamic nature of cognitive reserve, a corollary objective was to assess whether soft skills exhibit different associations with—proxies of—cognitive reserve that reflect an individual’s current lifestyle (at the time of the assessment) versus those that retrospectively depict their youth lifestyle.

Based on the literature, we hypothesize the following:

1. (Aim 1). Soft skills might represent a single factor in people from 50 to 80 years, as previously found with young adults (Casali et al. 2022; Feraco et al. 2022a, b).
2. (Aim 2). Soft skills are positively related to life satisfaction (Bruna et al. 2019) and cognitive reserve (Baumann et al. 2020; Bourgault 2019). Exploratorily, we investigated whether soft skills show a different relationship with current (at the time of assessment) and retrospective (as recalled from youth) cognitive reserve.
3. (Aim 3). We explored whether the relationship between soft skills and both life satisfaction and cognitive reserve changes from age 50 and older because some studies suggest that the effect of specific skills could change in older age (Baumann et al. 2020).

Since socio-demographic characteristics (gender) and cognitive functioning (fluid and crystallized intelligence) could influence our variables of interest (Boyle et al. 2021; Falzarano et al. 2022; Giacomucci et al. 2022; Stern 2009), these factors were controlled for in the analyses.

## Materials and methods

### Participants

A total of 479 volunteer Italian native speakers aged 50–80 years were involved in the study. Participants did not receive any compensation for their participation. A power analysis run via simulation suggested 400 participants sufficed to contemporarily detect the two main effects (positive association between soft skills and life satisfaction and cognitive reserve). The inclusion criteria were as follows: (a) a good health status assessed with a semi-structured interview (De Beni et al. 2008, p. 200); and (b) absence of signs of cognitive impairment, i.e., a score above 9 on the short version of the Italian checklist for the multi-dimensional assessment (SVAMA) of the elderly (Gallina et al. 2006). We excluded 37 participants because they did not achieve the minimum score on the SVAMA.

The final sample of 442 participants (*M*age = 64.07; *SD* = 9.37; 227 males) was evenly distributed across three age groups. See Table 1 for descriptive statistics of socio-demographic information by group.

**Table 1:** Descriptive statistics of the demographic characteristics by age groups

	Group 1 50–59 years: (*N* = 152; 76 males)		Group 2 60–69 years: (*N* = 150; 77 males)		Group 3 70+ years (*N* = 140; 74 males)	
	*M*	SD	*M*	SD	*M*	SD
Age	53.99	2.79	63.79	3.13	75.33	4.52
Education level*	4.42	1.25	4.81	1.26	4.32	1.41
Married	90%		92%		79%	
With children	86%		85%		88%	

*Education is measured on a 7-point scale with 1 = no school title; 4 = high school title; 7 = higher than master degree

The study was approved by the local ethics committee for psychological research (N: 3865) and performed according to the Helsinki Declaration.

### Materials

All measures showed good internal reliability (Cronbach’s alpha) as calculated for the study sample (see Table 2 in Results section).

**Table 2:** Mean, standard deviation, Cronbach’s alphas, and correlations between all the variables of interest

	*M*	SD	*α*	1	2	3	4	5	6	7
1. Age	64.07	9.37	–							
2. Soft skills	–	–	0.83	− 0.08						
3. Fluid intelligence	16.99	5.15	0.77	− 0.27	0.08					
4. Crystallized intelligence	31.35	7.99	0.76	− 0.06	0.13	0.39				
5. Life satisfaction	34.01	5.38	0.88	0.06	0.46	− 0.01	− 0.16			
6. Cognitive reserve	78.48	22.54	0.88	0.15	0.37	0.15	0.32	0.24		
7. CR-current	51.54	13.34	0.80	0.12	0.38	0.16	0.33	0.28	0.91	
8. CR-retrospective	26.94	11.71	0.83	0.14	0.27	0.10	0.23	0.15	0.89	0.62

CR, cognitive reserve. With 442 observations, all correlations >|0.09| are significant with *p* < 0.05; all correlations >|0.12| are significant with *p* < 0.01; all correlations >|0.15| are significant with *p* < 0.001. Mean and standard deviation are not meaningful for the soft skills latent score and are not reported

#### Soft skill measures

We used six measures for soft skills to cover the six skills included in the WEF model (World Economic Forum 2016).

*Adaptability Scale* (Feraco et al. 2023a, b). This scale measures the ability to adapt functionally to new and uncertain circumstances using nine items on a 7-point Likert scale (e.g., “I am able to adjust my thinking or expectations to assist me in a new situation”).

*Epistemic Curiosity Scale* (Litman 2008). This scale measures the desire for knowledge that motivates individuals to learn using five items on a 5-point Likert scale (e.g., “I enjoy exploring new ideas”).

*Personal Growth Initiative Scale* (Robitschek 1998). This scale measures one’s tendency toward proactively taking the initiative using nine items on a 7-point Likert scale (e.g., “I take charge of my life”).

*Leadership Scale* (Feraco et al. 2022a, b; Peterson and Seligman 2004). This scale measures one’s ability to take responsibilities for a group and lead others to success. Respondents provided answers to five items on a 5-point Likert scale (e.g., “My friends always tell me I am a strong but fair leader”).

*Short Grit Scale* (Duckworth and Quinn 2009; Sulla et al. 2018). This scale measures grit—that is, passion for long-term goals. We included only the four items referring to perseverance in effort (i.e., to maintain one’s effort despite difficulties: “Setbacks don’t discourage me”) in our analysis following previous review of the literature evidencing its prominence over the consistency of interest subdimensions (Credé et al. 2017). Participants responded using a 5-point Likert scale.

*Social and Cultural Awareness Scale* (Feraco et al. 2022a, b; Peterson and Seligman 2004). This scale measures one’s awareness of social interactions and culture. We used the five social intelligence items of the VIA-IS-120 inventory to measure social awareness (e.g., “I know how to handle myself in different social situations”) on a 5-point Likert scale. We used the items from the soft skills questionnaire (Feraco et al. 2022a, b) to measure cultural awareness (e.g., “I believe it’s important to respect the environment”) on a 6-point Likert scale.

We used latent scores to calculate the general soft skills’ score.

#### Measures for cognitive abilities

*Cattell Test – Scale 3A* (Cattell 1940). This measures fluid intelligence and consists of four timed (series, classifications, matrices, and conditions) subtests with a total of 60 items to complete. Participants receive one point for each correct answer and zero points for incorrect or missing answers. Total score is the number of correct answers.

*Primary Mental Abilities – Verbal* (Thurstone and Thurstone 1963). This task measures crystallized intelligence with 50 items. Each item consists of a target word and five alternatives, and the respondent had to select a synonym for the target word. The total score is the number of correct answers.

#### Life satisfaction and cognitive reserve

*Subjective Well-being Questionnaire – Personal Satisfaction Subscale* (De Beni et al. 2008). This questionnaire measures one’s satisfaction with his/her own life with 11 items (e.g., “I like how I am”) on a 4-point Likert scale. The score is calculated as the sum of the scores for all items, with higher scores indicating greater personal satisfaction.

*Current and Retrospective Cognitive Reserve survey* (2CR; Borella et al. 2023). It consists of groups of items spanning five dimensions of experience: socioeconomic status (e.g., educational level; occupational class); family engagement (e.g., partner’s status in terms of physical and mental health, partnership quality); leisure activity (e.g., engagement in recreational exercise, creative expression, and intellectual stimulation); social engagement (e.g., participation in volunteering, or public events); spiritual/religious practice (e.g., praying and attending religious rites/ceremonies). The three latter dimensions (leisure activity, social engagement, spiritual/religious practice) were assessed with respect to current status (CR-current, at the time of assessment) and retrospective status (CR-retrospective, referring to younger adulthood, when they were 20 to 35–40 years old), whereas socioeconomic status and family engagement were assessed with respect to current status only (CR-current, at the time of assessment). Participants reported the frequency of engagement in the different activities using a 5-point Likert scale (Borella et al. 2023). A global score is calculated as the sum of the scores for all current and retrospective dimensions, whereas two CR-current and CR-retrospective scores are computed as the sum of the scores on their related dimensions, with higher scores corresponding to greater cognitive reserve.

### Procedure

Data were collected remotely (due to the COVID-19 pandemic) between December 2021 and April 2022. The experimenters (i.e., six trained master’s students in psychology) contacted participants personally and invited them to participate in the study. After giving their informed consent, participants completed two Zoom meetings lasting about 60 min each. The experimenter sent a Qualtrics link via the Zoom chat, to complete the tasks and questionnaires. During the first meeting, participants provided personal information and completed the SVAMA, the Cattell, and the subjective well-being questionnaire. During the second meeting, they completed the 2CR, the soft skills questionnaires, and the PMA-Verbal. Measures were randomized except for the personal information and the SVAMA. The experimenter carefully explained the tasks and presented sample items. Data were completely anonymous, and the researchers had no access to information that could identify individual participants.

### Statistical analysis

We ran all analyses using the R software (R Core Team 2022; version 4.3.1) in R Studio (2023.12.1). First, for descriptive purposes we computed means and standard deviations of all considered variables and correlations between them. Then, statistical analysis followed the aims of the study.

For Aim 1, we could expect the six soft skills to converge in a single overarching factor (Casali et al. 2022; Feraco et al. 2023a, b). Therefore, we ran a confirmatory factor analysis including the measurement models of the single soft skills (i.e., adaptability, curiosity, initiative, leadership, perseverance, and social and cultural awareness are considered latent variables predicting observed scores in their corresponding items) and the overarching second-order factor that directly affects the latent first-order soft skills factors. We assessed the fit of the model using the comparative fit index (CFI), the Tucker–Lewis index (TLI), and the standardized root mean squared residual (SRMR) (Bentler and Bonett 1980). This index was selected over the more commonly used root mean squared error of approximation (RMSEA) because recent simulation studies demonstrated its greater reliability with ordinal data in large samples (Maydeu-Olivares et al. 2018; Shi et al. 2020). We considered the model to have a good fit when CFI and TLI values were 0.95 or more, and SRMR values were 0.09 or less (Bentler and Bonett 1980). We then obtained the latent score from the model to estimate a general soft skills’ score for each participant.

Then, to accomplish Aim 2, we fitted two sets of regression models to study the association between soft skills and the dependent variables of interest: life satisfaction and cognitive reserve (in its current and retrospective components). For each dependent variable, in the first model we added the following covariates as predictors: age, gender, and fluid and crystallized intelligence variables. We added soft skills in a second model to allow the calculation of the additional variance explained by soft skills (i.e., Δ*R*^2^). We also compared the models using the AIC and BIC indices and the likelihood ratio test. We ran additional analysis to study the association between soft skills and current and retrospective cognitive reserve and the effects of specific skills when the general factor of soft skills is taken into account.

To accomplish Aim 3, we added the interaction effect (soft skills × age) to the models to control whether the effect of soft skills on the two outcomes (life satisfaction and cognitive reserve) varies as a function of age. We compared the models using the AIC and BIC indices. If the two indices decrease when the interaction effect is added, evidence will favor such a model; otherwise, evidence will suggest that the interaction effect is not informative.

## Results

First, we computed descriptive statistics and correlations between all the variables of interest as shown in Table 2.

### Measurement of soft skills as a single overarching factor

The results of the measurement model (Aim 1) for soft skills were acceptable both at the first-order level (i.e., single soft skills) and at the second-order level (i.e., with an overarching factor of soft skills). For the complete model, fit indices were CFI = 0.96, TLI = 0.96, and SRMR = 0.08. For the complete set of fit indices, refer to Table 3 or Table S1 in supplementary material for the standardized loadings. All the loadings were significant with *p* < 0.001, and the mean loading for the second-order factor was 0.68. Therefore, the results confirmed, as hypothesized, that soft skills form a unique factor also in the age range considered here.

**Table 3:** Fit indices for the measurement models of soft skills

	*χ*^2^	*df*	CFI	TLI	SRMR
Adaptability	371.16	27.00	0.98	0.98	0.08
Curiosity	21.20	5.00	1.00	1.00	0.04
Initiative	141.14	27.00	0.99	0.99	0.05
Leadership	50.79	5.00	0.98	0.97	0.07
Perseverance	3.81	2.00	1.00	0.99	0.03
Social and cultural awareness	371.16	27.00	0.98	0.98	0.08
Soft skills (second-order model)	3703.62	854.00	0.96	0.96	0.08

CFI, comparative fit index; TLI, Tucker–Lewis index; SRMR, standardized root mean squared residual

Descriptive statistics for soft skills as well as correlations between unique soft skills factor and other variables are available in Supplementary materials (Table S2).

### Association between soft skills, life satisfaction, and cognitive reserve

The regression models (Aim 2) showed that soft skills, as a unique factor, have a positive association with both life satisfaction and cognitive reserve, as hypothesized. In fact, the likelihood ratio test was always significant (*p* < 0.001), and both the AIC and the BIC consistently decreased (see Table 4) and soft skills were positively associated with both life satisfaction (*β* = 0.54) and cognitive reserve (*β* = 0.40). In particular, compared to the baseline model without soft skills, soft skills additionally explained 23% of the variance in life satisfaction (total *R*^2^ = 28%) and 12% of the variance in cognitive reserve (total *R*^2^ = 26%). The complete set of results is reported in Table 4. The results were confirmed for both current and retrospective cognitive reserve; soft skills were in fact positively associated with both current (*β* = 0.41) and retrospective (*β* = 0.30) cognitive reserve. In particular, compared to the baseline model without soft skills, soft skills additionally explained 13% of the variance for current (total *R*^2^ = 26%) and 7% for retrospective (total *R*^2^ = 17%) cognitive reserve scores, respectively. The complete set of results is reported in Table 4.

**Table 4:** Complete results of the regression analyses

Predictor	Β	Se	*t*	ΔAIC	ΔBIC
*Regression on life satisfaction*				− 119	− 115
Soft skills	0.54***	0.05	11.73		
Fluid intelligence	0.07	0.05	1.60		
Crystallized intelligence	− 0.23***	0.04	− 5.25		
Age	0.01	0.00	2.27		
Gender-Female	− 0.26***	0.08	− 3.13		
*Regression on cognitive reserve*				− 65	− 61
Soft skills	0.40***	0.05	8.48		
Fluid intelligence	0.09	0.05	1.85		
Crystallized intelligence	0.25***	0.05	5.43		
Age	0.02***	0.00	5.01		
Gender-Female	0.17*	0.08	2.01		
*Regression on CR-current*				− 69	− 64
Soft skills	0.41***	0.05	8.68		
Fluid intelligence	0.08	0.05	1.78		
Crystallized intelligence	0.26***	0.05	5.76		
Age	0.02***	0.00	4.39		
Gender-Female	0.05	0.08	0.60		
*Regression on CR-retrospective*				− 34	− 30
Soft skills	0.30***	0.05	6.08		
Fluid intelligence	0.07	0.05	1.46		
Crystallized intelligence	0.18***	0.05	3.69		
Age	0.02***	0.00	4.40		
Gender-Female	0.26**	0.09	3.00		

ΔAIC and ΔBIC refer to the difference between the models with soft skills and the models without soft skills

*p < 0.01; **p < 0.01; ***p < 0.001

Gender was a dichotomous variable (0 = male; 1 = female)

For what concerns the covariates, in our sample females reported significantly lower life satisfaction and higher total and retrospective cognitive reserve; older people reported significantly higher cognitive reserve (total, current, and retrospective) and life satisfaction; crystallized intelligence was positively associated with cognitive reserve (total, current, and retrospective) and negatively with life satisfaction; fluid intelligence was not significantly associated with any outcome variables.

Additional analyses were conducted by incorporating the six specific soft skills after removing the variance explained by the general factor of soft skills. These analyses confirmed the significant role of the general factor, but also indicate that certain specific skills contribute to predict life satisfaction and cognitive reserve. Specifically, although adding the six soft skills explained less additional variance compared to the general factor alone (23% vs. 5% for life satisfaction and 12% vs. 8% for cognitive reserve), initiative (*β* = 0.40), and perseverance (*β* = 0.17) emerged as significant predictors for life satisfaction. For cognitive reserve, initiative (*β* = 0.26), social and cultural awareness (*β* = 0.44), and curiosity (*β* = 0.20) were significant predictors. The complete set of results is detailed in Table S3 in the Supplementary Materials.

### The effect of age in the relationship between soft skills, cognitive reserve, and life satisfaction

All the models without the interaction effect between soft skills and age, including the models with current and retrospective cognitive reserve, resulted preferable compared to the models with the interaction effect (Aim 3). We reached this conclusion using the likelihood ratio test and by comparing the AIC and BIC indices of the models with and without the interactions. In particular, the likelihood ratio test was never significant (*p* > 0.05), the AIC index increases of 0 and 1 points and the BIC of 4 and 5 points, respectively, in the life satisfaction and current reserve models with the interaction. Additionally, the interaction effect was never significant (*p* > 0.05). For these reasons and based on the models without interaction obtained in the previous section (Table 4), we suggest that there is an effect of soft skills on life satisfaction and cognitive reserve that does not vary as a function of age.

Overall, the effect of soft skills on life satisfaction and cognitive reserve was positive and did not change across the entire age span considered, even if age seems to play a role for cognitive reserve. Similar results were obtained for current and retrospective cognitive reserve.

## Discussion and conclusions

This study newly contributes to elucidating the nature and role of soft skills in middle adulthood and older age (ranging from 50 to 80 years) by referencing an established framework of soft skills (WEF 2016). Within this specific age range, we explored the relationships of these soft skills with two well-known indicators and core aspects of healthy aging: life satisfaction and—current and retrospective—cognitive reserve.

First of all (Aim 1), our results showed that soft skills can be considered together as a general factor. This means that soft skills not only in young adults (Casali et al. 2022; Feraco et al. 2023a, b), but also in middle adulthood and older age, and even if composed by different dimensions (including social, emotional, and cognitive aspects) should be also considered as a unidimensional factor, because specific skills might not have a role beyond it (Feraco et al. 2023a, b). Indeed, this unique factor is related to important outcomes in older age too, as highlighted by the regression analyses. Although this factor explained more variance in both outcomes, further analyses revealed that certain individual soft skills also accounted for additional variance. Such a pattern of findings highlight that soft skills also have a specific role depending on the outcome considered (i.e., initiative resulted positively associated with both life satisfaction and cognitive reserve, while perseverance, cultural awareness, and curiosity were significantly related to life satisfaction or cognitive reserve only). Therefore, our results suggest that soft skills can be considered at both a general and at an individual level to fully understand their overall impact and the specific effects each one exerts. Researchers are thus encouraged to carefully select soft skills to measure both their general factor and the specific skills of interest.

Concerning the associations with life satisfaction and cognitive reserve (Aim 2), the findings support the notion that soft skills may play a role in sustaining individuals across various aspects of their lives that are important for their autonomy and well-being in middle and late adulthood. In fact, multiple regression analyses revealed a positive relationship between soft skills and the outcome variables—above and beyond socio-demographics and fluid and crystalized intelligence.

Soft skills contributed to explain a substantial portion of the variance in participants’ life satisfaction, consistent with research emphasizing soft skills and other character qualities as crucial contributors to subjective well-being (Bruna et al. 2019; Schutte and Malouff 2019). Indeed, soft skills are believed to propel individuals toward adopting a more positive approach to life’s diverse challenges (Park et al. 2004). Our results align with this concept and innovatively extend it to middle adulthood and older age, periods that have received limited attention. They indeed suggest that such individual qualities might represent resources that individuals deploy to regulate thoughts, emotions, and behaviors in a variety of life contexts also in later life stages, thereby feeling satisfied with themselves, balanced and self-realized as well as capable of coping with future challenges and opportunities. According to this line of reasoning, perseverance and initiative emerged as specific positive predictors of life satisfaction, supporting the idea that taking initiative and persevering toward ones aims would be particularly important for life satisfaction in older ages.

The role of soft skills in association with life satisfaction clearly emerged above and beyond the modest contribution of some of the socio-demographic characteristics and cognitive measures here controlled. Men demonstrated higher life satisfaction than women, suggesting that gender, as shown in other studies, may be one of the socio-demographic characteristics likely to shape individuals’ opportunities and priorities, consequently influencing their well-being (Joshanloo 2018). There was also a surprising negative association between the measure of crystallized intelligence used and life satisfaction. This result, which warrants further exploration, appears to align with prior findings arguing that individuals with higher crystallized intelligence may engage more in challenging contexts and work/social environments of everyday life, potentially encountering more daily stressors and consequently reporting reduced perceived well-being and satisfaction with their lives (see Falzarano et al. 2022). Fluid intelligence (reasoning abilities measured by the Cattell test) did not exhibit an association with life satisfaction; this result is consistent with previous findings indicating that fluid abilities predict life satisfaction in younger ages but not in older adults, possibly because they are more important for job performance in younger ages (Falzarano et al. 2022).

Our study revealed a novel positive—though modest—association also between soft skills and cognitive reserve. This finding aligns with prior evidence of the connections between such individual qualities with extracurricular activities in younger age (Feraco et al. 2022a, b; Park et al. 2004; Robles 2012) and confirms that these qualities are correlated with socio-behavioral indicators of a stimulating lifestyle. This includes engagement in a variety of intellectual, leisure, and social activities, which are well-known protective factors contributing to successful and healthy aging. In essence, the way soft skills contribute to regulate thoughts, emotions, and behaviors is linked to positive lifestyles that may safeguard individuals from cognitive decline and enhance overall well-being. Interestingly, initiative, curiosity, and social and cultural awareness explained additional specific variance, suggesting that people who take initiative and have higher interest toward novelty and social issues might be particularly prone to engage in stimulating and enriching activities able to sustain their cognitive reserve in older age. Deeply exploring the associations between soft skills and the current and retrospective cognitive reserve scores, our results indicated that soft skills appear to have a greater, but modest, impact in explaining current rather than retrospective cognitive reserve (as indicated by larger variance explained in the first respect to the latter). Such a result further suggests the importance of regarding cognitive reserve as a dynamic, life stage-dependent construct, continuously molded as adults adapt to changing demands, goals, priorities, and opportunities throughout life (see Borella et al. 2023).

It is worth mentioning that soft skills not only explained a greater portion of the variance in life satisfaction than in cognitive reserve, but also contributed to explain cognitive reserve, and its current and retrospective scores, to an equal extent as some of the other socio-demographic characteristics and cognitive measures considered. In particular, our results showed that age exhibited a—modest—association with cognitive reserve and its dimensions, aligning with the understanding that this protective factor accumulates over the life course (Stern 2009). Women displayed a higher retrospective cognitive reserve than men, suggesting how gender differences might also impact individuals’ opportunities and priorities in this area as well, thereby influencing their lifestyles (Giacomucci et al. 2022). Finally, crystallized intelligence, but not fluid intelligence, exhibited a positive association with cognitive reserve, further underscoring that cognitive reserve is more strongly connected to well-preserved cognitive skills, such as crystallized intelligence (Boyle et al. 2021).

Nonetheless, this first attempt to capture the link between soft skills and cognitive reserve does suggest that the way in which soft skills regulate thoughts, emotions, and behaviors is linked to and might contribute to shape individuals’ lifestyles that may safeguard them from cognitive decline. Our results and speculations deserve to be further deepened in future studies, also considering older individuals with different cognitive functioning profiles to better capture and disentangle the link between such individual qualities and proxies of cognitive reserve.

For Aim 3, our exploratory results showed that individuals of different ages benefit similarly from their soft skills, displaying a consistent pattern of relationships between soft skills, life satisfaction, and cognitive reserve (both retrospective and current). This finding appears to challenge the notion that the impact of soft skills on life satisfaction and proxies of cognitive reserve may slightly decrease with age, as suggested by Baumann et al. (2020). It is important to note, however, that there are differences in the way in which both soft skills and cognitive reserve were operationalized in the study by Baumann et al. (2020) and the present one that might explain such contrasting findings. Baumann et al. (2020) focused on the specificity of skills rather than on their joint contribution and considered only some specific proxies of cognitive reserve related to family engagement and partnership. Our results therefore suggest that soft skills could be regarded as enduring qualities that, despite undergoing—age-related—changes, continue to predict life satisfaction and other protective factors, such as cognitive reserve, throughout the adult lifespan and even in later life stages. The sustained relevance of soft skills in later stages of life underscores the importance of promoting these skills through targeted interventions. Nonetheless, these results should be considered preliminary, and more research is needed to directly replicate and test our results.

While our study provides a novel examination of the association between significant personal factors such as soft skills and key aspects of aging in a large sample of middle-aged and older adults, it comes with certain limitations. We employed a cross-sectional approach, preventing us from testing the causality of the effects or studying within-person variability over time. Our sample also comprises typically aging adults and older adults with a good health status and an active and engaged lifestyle, an aspect that might have influenced our results. Future studies are encouraged to further investigate and extend the results to people at risk of developing pathological aging trajectories or health issues. Additionally, all measures—soft skills, life satisfaction, and cognitive reserve—relied on self-reported data, and common-method variance may partly account for their associations. Additionally, cognitive reserve was self-reported and retrospectively measured, and only few broad measures of cognitive functions were employed, limiting our ability to precisely capture participants’ cognitive functioning and their impact on soft skills. Future studies could enhance precision by adopting a multi-method approach or incorporating other-reported measures alongside self-reported ones. This would contribute to a more comprehensive understanding of the variables under investigation.

In conclusion, while soft skills are traditionally seen as crucial factors for success and well-being in educational and work contexts (WEF 2016), our results expand their significance by highlighting their role in sustaining subjective well-being, specifically in terms of life satisfaction, and their contribution to explain also cognitive reserve, into adulthood—encompassing both middle-aged and older adults. Notably, their impact on life satisfaction and cognitive reserve remained consistent through age, suggesting that they serve as positive psychological resources throughout the life course. Although additional research is warranted, our findings underscore the importance of promoting also soft skills. This can aid individuals in engaging in lifestyle-related socio-behavioral experiences that contribute to healthy aging.

## Supplementary Information

Below is the link to the electronic supplementary material.

Supplementary file1 (DOCX 34 KB)

## Data Availability

The data that support the findings of this study are openly available in figshare at https://figshare.com/s/39070c009fa5f8350a04. Materials and code are available from the corresponding authors on request.
