# Supplementary material for: Soft skills and their relationship with life satisfaction and cognitive reserve in adulthood and older age
Source: Eur J Ageing. 2024 Sep 9;21(1):25. doi: 10.1007/s10433-024-00820-2 (PMC11383892; doi:10.1007/s10433-024-00820-2)
Supplement: Supplementary file 1 — Supplementary file1 (DOCX 34 KB) [file 10433_2024_820_MOESM1_ESM.docx]

# Supplementary materials

**Soft skills and their relationship with cognitive reserve and life satisfaction in adulthood and older age**

**Table S1.** Standardized loadings of the CFA model of soft skills

| Latent variable | Op | Lower order variable | Loading |
| --- | --- | --- | --- |
| Soft skills | → | Adaptability | .78 |
| Soft skills | → | Curiosity | .73 |
| Soft skills | → | Perseverance | .57 |
| Soft skills | → | Initiative | .78 |
| Soft skills | → | Leadership | .50 |
| Soft skills | → | Social and cultural awareness | .74 |
| Adaptability | → | A_1 | .81 |
| Adaptability | → | A_2 | .83 |
| Adaptability | → | A_3 | .81 |
| Adaptability | → | A_4 | .81 |
| Adaptability | → | A_5 | .82 |
| Adaptability | → | A_6 | .73 |
| Adaptability | → | A_7 | .76 |
| Adaptability | → | A_8 | .75 |
| Adaptability | → | A_9 | .77 |
| Curiosity | → | CU_1 | .78 |
| Curiosity | → | CU_2 | .91 |
| Curiosity | → | CU_3 | .91 |
| Curiosity | → | CU_4 | .81 |
| Curiosity | → | CU_5 | .71 |
| Perseverance | → | G_2 | .10 |
| Perseverance | → | G_4 | .58 |
| Perseverance | → | G_7 | .80 |
| Perseverance | → | G_8 | .69 |
| Initiative | → | I_1 | .77 |
| Initiative | → | I_2 | .76 |
| Initiative | → | I_3 | .70 |
| Initiative | → | I_4 | .69 |
| Initiative | → | I_5 | .87 |
| Initiative | → | I_6 | .80 |
| Initiative | → | I_7 | .68 |
| Initiative | → | I_8 | .68 |
| Initiative | → | I_9 | .73 |
| Leadership | → | L_1 | .69 |
| Leadership | → | L_2 | .87 |
| Leadership | → | L_3 | .71 |
| Leadership | → | L_4 | .66 |
| Leadership | → | L_5 | .74 |
| Social and cultural awareness | → | SA_1 | .62 |
| Social and cultural awareness | → | SA_2 | .68 |
| Social and cultural awareness | → | SA_3 | .56 |
| Social and cultural awareness | → | SA_4 | .59 |
| Social and cultural awareness | → | SA_5 | .61 |
| Social and cultural awareness | → | CA_1 | .27 |
| Social and cultural awareness | → | CA_2 | .45 |
| Social and cultural awareness | → | CA_3 | .48 |
| Social and cultural awareness | → | CA_4 | .71 |
| Social and cultural awareness | → | CA_5 | .65 |
| Social and cultural awareness | → | CA_6 | .78 |

*Note.* A = Adaptability item; CU = Curiosity item; G = Perseverance item; I = Initiative item; L = Leadership item; SA = Social awareness item; CA = Cultural awareness item

**Table S2.** Descriptive statistics and correlations between single soft skills and other study variables

|  | M | SD | .1 | .2 | .3 | .4 | .5 | .6 | .7 |
| --- | --- | --- | --- | --- | --- | --- | --- | --- | --- |
| 1.Age | 64.07 | 9.37 | 1 |  |  |  |  |  |  |
| 2.Adaptability | 46.44 | 6.99 | -09 | 1 |  |  |  |  |  |
| 3.Curiosity | 20.19 | 3.48 | -07 | 52 | 1 |  |  |  |  |
| 4.Initiative | 44.29 | 7.08 | -04 | 60 | 44 | 1 |  |  |  |
| 5.Leadership | 19.15 | 3.47 | 06 | 22 | 29 | 26 | 1 |  |  |
| 6.Perseverance | 14.72 | 2.37 | 04 | 28 | 26 | 33 | 22 | 1 |  |
| 7.Social and cultural awareness | 9.03 | 92 | -07 | 48 | 54 | 47 | 39 | 25 | 1 |
| 8.Fluid intelligence | 16.99 | 5.15 | -27 | 14 | 09 | 01 | 01 | 07 | 08 |
| 9.Crystallized intelligence | 31.35 | 7.99 | -06 | 15 | 16 | 01 | 01 | 02 | 20 |
| 10.Life satisfaction | 34.01 | 5.38 | 06 | 37 | 26 | 50 | 14 | 31 | 27 |
| 11.Cognitive Reserve | 78.48 | 22.54 | 15 | 24 | 32 | 31 | 16 | 12 | 44 |
| 12.CR-current | 51.54 | 13.34 | 12 | 26 | 32 | 35 | 13 | 15 | 42 |
| 13.CR-retrospective | 26.94 | 11.71 | 14 | 16 | 25 | 20 | 16 | 06 | 38 |

*Note.* Social and cultural awareness is calculated as the sum of the average score in social awareness and cultural awareness subscales

**Table S3.** Complete results of the regression analyses including the specific soft skills as predictors.

| Predictor | Β | Se | t |
| --- | --- | --- | --- |
| **Regression on life satisfaction** |  |  |  |
| Soft skills (general factor) | .54*** | .05 | 12.05 |
| Fluid intelligence | .06 | .05 | 1.60 |
| Crystallized intelligence | -.21*** | .04 | -5.25 |
| Age | .01* | .00 | 2.27 |
| Gender-Female | -.22** | .08 | -3.13 |
| Adaptability | .17 | .10 | 1.74 |
| Curiosity | .05 | .08 | 0.67 |
| Initiative | .40*** | .10 | 4.08 |
| Leadership | -.03 | .05 | -.67 |
| Perseverance | .17** | .05 | 3.21 |
| Social and cultural awareness | .11 | .08 | 1.44 |
| **Regression on cognitive reserve** |  |  |  |
| Soft skills (general factor) | .40*** | .04 | 8.98 |
| Fluid intelligence | .10* | .05 | 2.13 |
| Crystallized intelligence | .21*** | .04 | 4.81 |
| Age | .02*** | .00 | 5.27 |
| Gender-Female | .17* | .08 | 2.12 |
| Adaptability | .05 | .10 | 0,52 |
| Curiosity | .20** | .08 | 2.64 |
| Initiative | .36*** | .10 | 3.58 |
| Leadership | -.00 | .05 | -.08 |
| Perseverance | .01 | .05 | 0.24 |
| Social and cultural awareness | .43*** | .08 | 5.52 |
